# Halofuginone down-regulates Smad3 expression and inhibits the TGFbeta-induced expression of fibrotic markers in human corneal fibroblasts

**Published:** 2012-02-18

**Authors:** Elizabeth F. Nelson, Craig W. Huang, Jillian M. Ewel, Angela A. Chang, Ching Yuan

**Affiliations:** Department of Ophthalmology, University of Minnesota, Minneapolis, MN

## Abstract

**Purpose:**

Due to its ability to disrupt transforming growth factor beta (TGF-β) signaling, halofuginone has been successfully used to treat various fibrotic disorders. Here we investigated the antifibrotic potential of halofuginone in human corneal fibroblasts.

**Methods:**

Human corneal fibroblasts were isolated from human donor corneas for in vitro experiments. TGF-β was used to stimulate pro-fibrotic responses from corneal fibroblasts under halofuginone treatment. The expression of alpha smooth muscle actin (α-SMA) and fibronectin was analyzed by western blots. Phalloidin toxin was used to stain cultures for stress fiber assemblies. Quantitative reverse transcription PCR (qRT–PCR) and immunostaining were used to analyze the expression of type I collagen mRNA and protein, respectively. The expression of Smad2, Smad3, phospho-Smad2, and phospho-Smad3 was determined by western blots.

**Results:**

Halofuginone was well tolerated by human corneal fibroblasts up to 10 ng/ml as demonstrated by a cell viability assay. At this concentration, TGF-β-induced expression of the fibrotic markers α-SMA, fibronectin, and type I collagen was significantly reduced. Interestingly, under our experimental conditions, halofuginone treatment led to reduced protein expression of Smad3, which was both dose- and time-dependent.

**Conclusions:**

Our results suggest that halofuginone may exert its antifibrotic effects in the cornea via a novel molecular mechanism and may be used as an antifibrotic agent for corneal fibrosis treatment.

## Introduction

Fibrotic disorders affect all tissues and organs. Although fibrosis is an important tissue repair mechanism, it can negatively impact the integrity of a tissue if it progresses excessively. The fibrotic process involves multiple signaling pathways, which activate myofibroblast transformation, fibrotic marker expression, and extracellular matrix (ECM) protein deposits. Among these pro-fibrotic signaling pathways, transforming growth factor beta (TGF-β) is likely the most important as demonstrated by in vitro experiments and animal models [1-3]. The TGF-β signal is transduced through TGF-β receptor type I (TβR-I, also known as ALK5) and type II (TβR-II, a serine/threonine protein kinase) [4,5]. Binding of TGF-β induces the association of TβR-I and TβR-II, allowing TβR-II to phosphorylate the GS domain of TβR-I. Essential downstream effectors, including Smad proteins and mitogen-activated protein kinase (MAPK), are then activated by the phosphorylated receptor complex [6]. In Smad signaling, the phosphorylated receptor complex recruits receptor-regulated Smad proteins (R-Smad), including Smad2 and Smad3, and activates them by phosphorylation. The activated R-Smads form heterodimers and further complex with Smad4 (Co-Smad), then translocate into the nucleus to regulate transcription. Other non-Smad signaling pathways, including Rho/Rho-associated protein kinase (ROCK), MAP kinases, phosphatase 2A, signal transducers and activators of transcription/nuclear factor kappa-B (NFκB), and phosphatidylinositol-3 kinase (PI3K)/Akt, can also cross-talk with Smad signaling and therefore modulate the fibrotic processes [7].

In corneal tissue, fibrosis following injuries and surgeries or resulting from disorders, such as limbal stem cell deficiency, can cause corneal opacity and scarring, thereby compromising vision. Various researchers have confirmed the essential role of TGF-β signaling in corneal fibrosis, and thus vigorous investigation into preventing corneal fibrosis has focused on strategies to disrupt this pathway [8]. For example, overexpression of Smad7 by a gene therapy approach significantly reduced corneal fibrosis in an animal model [8,9]. Chemical inhibitors targeting various pathways (indicated in parentheses), such as trichostatin A (histone deacetylase) [10], C3 transferase or Y27632 (Rho/ROCK) [11], and pioglitazone (peroxisome proliferator-activated receptor-gamma) [12] have also shown great promise in inhibiting the expression of fibrotic markers in vitro and/or suppressing fibrosis in animal models.

Halofuginone (7-bromo-6-chloro-3-[3-(3-hydroxy-2-piperidinyl)-2-oxopropyl]-4(3H)-quinazolinone) is a synthetic derivative of febrifugine, a plant alkaloid isolated from the roots of *Dichroa febrifuga*. Halofuginone has been used widely in the poultry industry for treating coccidiosis and has recently been applied for treating fibrosis and neovascularization and for anti-tumor therapy [13-15]. Both in vitro and animal studies have revealed that halofuginone effectively inhibits type I collagen synthesis in many cell types and tissues subjected to various injuries or stresses [16,17]. In dermal fibroblasts, halofuginone treatment not only significantly reduces the production of type I collagen, but also inhibits the expression of other fibrotic markers and ECM proteins [18]. The transformation of fibroblasts to myofibroblasts is also inhibited by halofuginone [19]. In experimental animal models of systemic sclerosis [20], chronic graft-versus-host disease [21] and radiation damage [22], halofuginone significantly reduces fibrosis, scarring and inflammation. Halofuginone has been approved by the United States Food and Drug Administration for treating scleroderma (Tempostatin^TM^; Collgard Biopharmaceutical Ltd., Petah Tikva, Israel).

The potent antifibrotic efficacy of halofuginone and its long history of safe usage in veterinary and clinical fields have prompted us to investigate its potential in preventing corneal fibrosis. Using corneal fibroblasts isolated from human donors, we have demonstrated that halofuginone has low cytotoxicity but high potency in suppressing the fibrotic markers alpha smooth muscle actin (α-SMA), fibronectin, and type I collagen. Our results also indicate that halofuginone exerts its antifibrotic effect in human corneal fibroblasts by down-regulating Smad3 protein expression, which has not been reported previously. Based on these preliminary results, we propose that halofuginone bears great promise for ophthalmic antifibrotic applications and warrants further study in animal models.

## Methods

### Human corneal fibroblast cultures

Human corneal fibroblasts were isolated from Institutional Review Board exempt donor tissue obtained from the Minnesota Lions Eye Bank (Minneapolis, MN). Central corneas were dissected from donor cornea buttons for overnight collagenase digestion [23]; collagenase I was purchased from Invitrogen (Carlsbad, CA). The digested samples were passed through a cell strainer to remove any pieces of undigested tissue, and cells were spun down and rinsed with DMEM (Invitrogen). Cells were propagated in fibroblast medium, which consists of a basal medium supplemented with 2% FBS, 1× fibroblast growth supplement and antibiotics and which is formulated specifically for the optimal growth and minimal differentiation of fibroblasts (ScienCell Research Laboratories, Carlsbad, CA). For halofuginone and TGF-β experiments, cells from passages 3–6 were cultured in DMEM supplemented with 10% FBS. Unless otherwise noted, cells were seeded at a density of approximately 10^4^ cells/cm^2^.

### Cytotoxicity of halofuginone

Human fibroblasts were seeded 3,000 cells per well in 96-well plates and grown to 30%–40% confluence. Cells were then treated with halofuginone (Toronto Research Chemicals, North York, Ontario, Canada) or vehicle. Twenty-four hours following the addition of halofuginone, 50 µM resazurin dye (Sigma-Aldrich, Saint Louis, MO) was added, and cells were allowed to incubate another 24 h. Resazurin fluorescence was then measured using an Fmax plate reader and instrument-specific software, SOFTmax PRO version 1.3.1 (Molecular Devices, Sunnyvale, CA). To determine baseline fluorescence, 0.1% Triton X-100 was used to lyse cells.

### Western blot experiments

For fibrotic marker experiments, human corneal fibroblasts were treated for one hour with 10 ng/ml halofuginone before adding 2 ng/ml TGF-β (Cell Sciences, Canton, MA), and cell lysates were harvested at 48 h. For Smad signaling experiments, human corneal fibroblasts were treated with 10 ng/ml halofuginone for 24 h, then 2 ng/ml TGF-β was added, and cell lysates were harvested at 30 min. Lysates were separated on 12% SDS–PAGE gels then transferred onto nitrocellulose membranes. Antibodies against fibrotic markers α-SMA (clone 1A4) and human fibronectin were purchased from Sigma-Aldrich, as well as antibody against β-actin (AC-15). Antibodies against phospho-Smad3 (Ser423/425; C25A9), phospho-Smad2 (Ser465/467; 138D4), and Smad2 (L16D3) were purchased from Cell Signaling Technology (Danvers, MA); and Smad3 (FL-425) and Smad7 (Z8-B) antibodies were purchased from Santa Cruz Biotechnology (Santa Cruz, CA). Antibody against GAPDH was purchased from Biodesign International (Saco, ME). Secondary antibodies labeled with IRDye 700 or IRDye 800 were purchased from Rockland Immunochemicals Inc. (Gilbertsville, PA). An Odyssey Infrared Imaging System (Li-Cor Biosciences, Lincoln, NE) was used to image protein bands, and included analysis software was used to digitize and convert data. Data were normalized to β-actin.

### qRT–PCR

Human corneal fibroblasts were treated for 1 h with 10 ng/ml halofuginone before the addition of 2 ng/ml TGF-β. Forty-eight hours after adding TGF-β, cells were lysed and total RNA was collected using an RNeasy Mini Kit (QIAGEN Inc., Valencia, CA); genomic DNA was digested with RNase-free DNase I (QIAGEN). Total RNA (5 µg) was then reverse transcribed using SuperScript III First Strand Synthesis System (Invitrogen). Quantitative reverse transcription PCR (qRT-PCR) was performed using an iCycler thermal cycler (Bio-Rad Laboratories, Inc., Hercules, CA) with iQ SYBR Green Supermix (Bio-Rad Laboratories) and primers for type I collagen alpha 2 (*COL1A2*; F, 5′-GCA CAT GCC GTG ACT TGA GA-3′; R, 5′-CAT CCA TAG TGC ATC CTT GAT TAG G-3′) [24] and β-actin (*ACTB*; F, 5′-CTG GCA CCC AGC ACA ATG-3′; R, 5′-AGC GAG GCC AGG ATG GA-3′) [25]. Thirty-five amplification cycles, consisting of 10 s at 95 °C and 30 s at 56 °C, were run on 25 µl reactions. A standard curve to determine the amplification efficiency (E) of each primer pair was generated from a series of cDNA log dilutions, where E=10^-1/slope of standard curve^. An iQ5 real-time PCR detection system and accompanying iQ5 optical system software (Bio-Rad Laboratories) were used to collect and analyze real-time PCR data; the fold change expression of *COL1A2* mRNA was normalized to *ACTB* mRNA by the Pfaffl method, taking into account the actual amplification efficiency of primer pairs.

### Immunostainings and phalloidin toxin staining

Human corneal fibroblasts were seeded in glass 8-well chamber slides, then treated for 1 h with 10 ng/ml halofuginone before adding 2 ng/ml TGF-β. Forty-eight hours after the addition of TGF-β, cells were fixed in 4% paraformaldehyde. For detection of type I collagen, cells were blocked with 10% rabbit normal serum in 1× PBS plus 0.02% Tween-20 then stained with rabbit anti-type I collagen antibody (1:20; Southern Biotech, Birmingham, AL) overnight at 4 °C. A rabbit anti-goat Alexa Fluor 488 (1:1,000; Invitrogen) was used as the secondary antibody. For phalloidin staining, cells were permeablized with 1% Triton X-100 following fixation, blocked in 1% BSA in 1× PBS, then incubated in Alexa Fluor 594 phalloidin toxin (1:40; Invitrogen). Nuclei were counterstained with Hoechst’s dye 33342. Cells were imaged with a Zeiss Axiovert 200M epi-fluorescent microscope (Carl Zeiss, Jena, Germany).

### Luciferase assay

Plasmids p3TP-Lux (a generous gift from Dr. Joan Massague, Memorial Sloan-Kettering Cancer Center, New York, NY) and pRL (Promega, Madison, WI) were transfected into human corneal fibroblasts with Fugene 6 (Roche Applied Science, Indianopolis, IN) according to the manufacturer’s instructions. p3TP-Lux contains the firefly luciferase gene whose expression is driven by a synthetic promoter containing three tandem repeats of the activator protein-1 binding motif and part of the plasminogen activator inhibitor-1 promoter, which is activated by Smad3 binding [26]; the *Renilla* luciferase encoded by pRL is constitutively expressed. Ten ng/ml halofuginone was added to cells 4 h after transfection, and 2 ng/ml TGF-β was added 24 h after transfection. Twenty-four hours after the addition of TGF-β (48 h after transfection), cell lysates were harvested and assayed with a Dual-Luciferase Reporter System Assay kit (Promega); the luciferase activity of lysates was measured with a Lumat LB 9507 Luminometer (Berthold, Germany). The respective activity of firefly luciferase and *Renilla* luciferase were measured sequentially from a single sample, and the ratio of reporter firefly luciferase to control *Renilla* luciferase was used for comparison.

## Results

### Cytotoxicity of halofuginone in corneal fibroblasts

**Figure 1:**
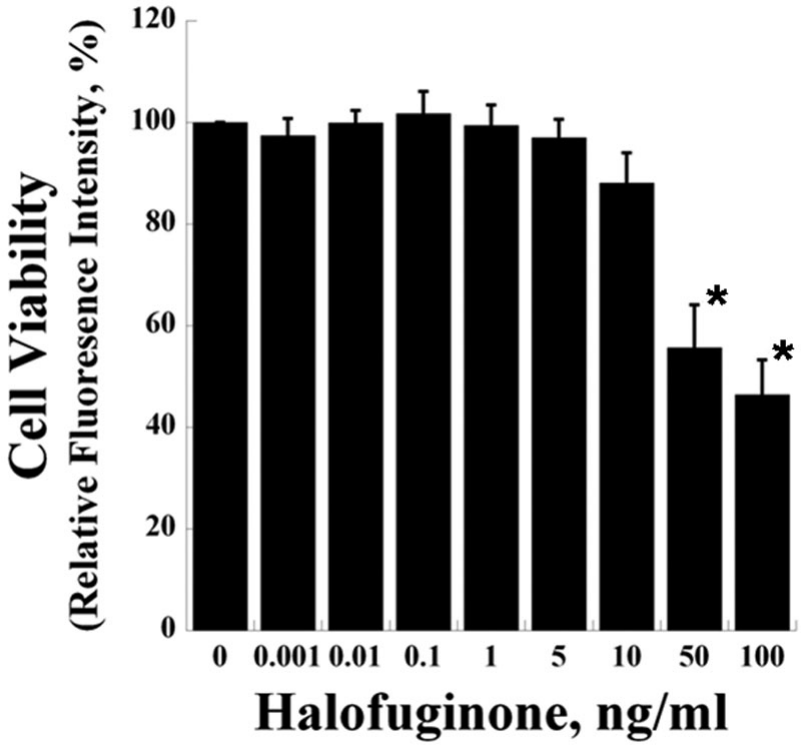
Halofuginone is nontoxic at low concentrations. The viability of human corneal fibroblasts treated with halofuginone is similar to control for concentrations of 10 ng/ml or less. The viability of cells treated with 50 ng/ml or 100 ng/ml halofuginone is significantly decreased compared to control (* p<0.005). Viability is expressed as fluorescence intensity of metabolized resazurin relative to control (n=5, bar=S.E.M.).

To determine the suitable treatment dosage of halofuginone, we assayed resazurin metabolism of human corneal fibroblasts treated with varying halofuginone concentrations. Halofuginone concentrations up to 10 ng/ml showed no significant toxicity compared to vehicle (Figure 1). However, at higher concentrations of 50 ng/ml and 100 ng/ml, halofuginone resulted in significantly decreased cell viability. Therefore, 10 ng/ml was chosen as the treatment dose for subsequent experiments.

### Halofuginone suppresses expression of α-SMA and fibronectin in corneal fibroblasts

**Figure 2:**
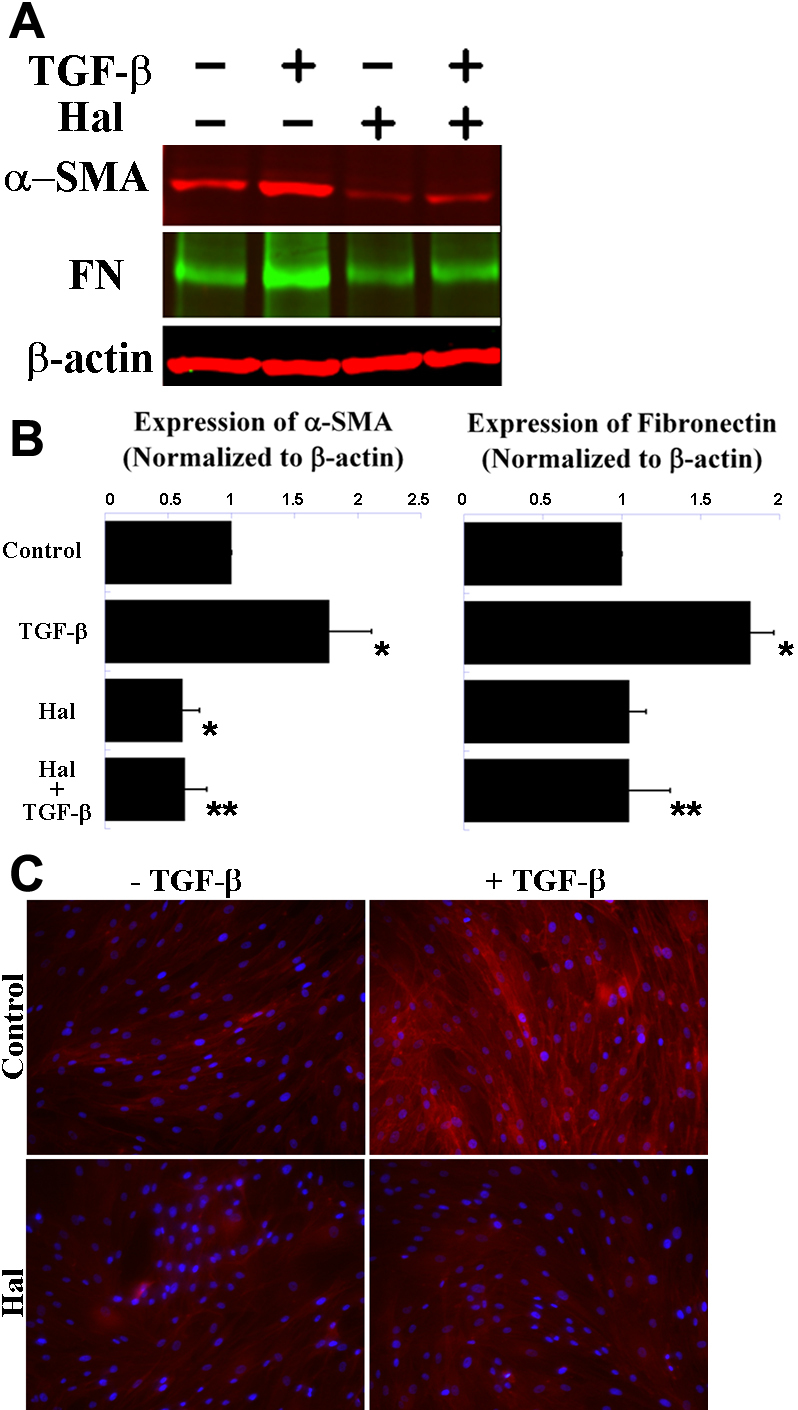
Halofuginone suppresses TGF-β-induced expression of fibrotic markers and stress fiber assemblies in human corneal fibroblasts. **A**: Representative western blots show increased expression of α-SMA and fibronectin (FN) after TGF-β treatment and inhibition of the induced expression by halofuginone (Hal). β-actin shown as loading control. **B**: Quantification and normalization of α-SMA and fibronectin western blot data. * p<0.05 compared to control, ** p<0.05 compared to TGF-β (n=4, bar=S.E.M.). **C**: Representative images of phalloidin toxin staining show cells treated with TGF-β (top right) display greater fluorescence signal than untreated control (top left) or cells treated with TGF-β and halofuginone (lower right).

In the fibrotic process, α-SMA and fibronectin are known to increase significantly in fibroblasts and have been commonly used as fibrotic markers. Western blot analysis confirmed that TGF-β treatment increased the expression of α-SMA and fibronectin in human corneal fibroblast cultures compared to no treatment (Figure 2A). Halofuginone treatment suppressed TGF-β-induced expression of α-SMA and fibronectin, as well as reduced the basal level of α-SMA (Figure 2A). Results from four independent experiments are summarized in Figure 2B.

### Halofuginone inhibits stress fiber assembly in corneal fibroblasts

Because α-SMA is a component of stress fibers, we investigated stress fiber assembly in human corneal fibroblasts by staining with fluorescent-labeled phalloidin toxin; we confirmed the positive regulation of stress fiber assembly by TGF-β (Figure 2C). In contrast, phalloidin toxin staining of cells co-treated with halofuginone was not only significantly less than the staining of cells treated with TGF-β alone, but was also similar to the staining of the untreated control (Figure 2C).

### Halofuginone inhibits type I collagen expression in corneal fibroblasts

**Figure 3:**
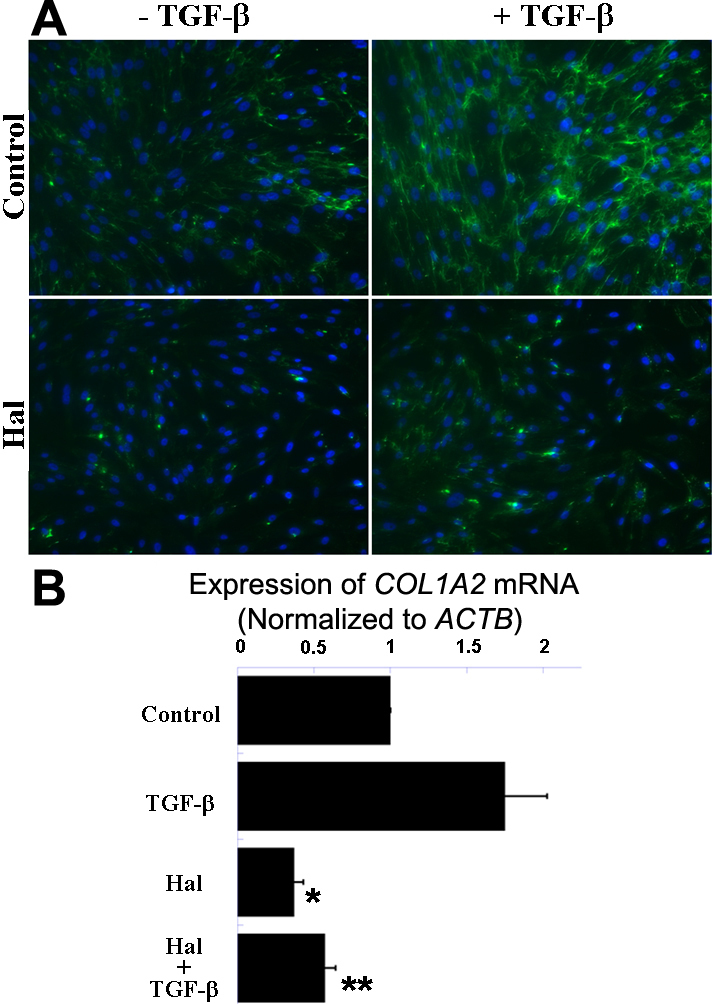
Halofuginone suppresses TGF-β-induced expression of type I collagen protein and mRNA in human corneal fibroblasts. **A**: Representative images of immunostainings for type I collagen show cells treated with halofuginone (Hal) and TGF-β (bottom right) display similar fluorescence signal as untreated control cells (top left), but less signal than cells treated with TGF-β only (top right). **B**: qRT–PCR analysis demonstrates treatment with halofuginone significantly reduces both TGF-β-induced expression and basal expression of *COL1A2* mRNA (normalized to *ACTB* mRNA). * p<0.05 compared to control, ** p<0.05 compared to TGF-β (n=4, bar=S.E.M.).

We also investigated the effect of halofuginone on the expression of another ECM protein and classic fibrotic marker, type I collagen. Human corneal fibroblasts co-treated with halofuginone and TGF-β displayed much weaker immunostaining signals for type I collagen than cells stimulated with TGF-β alone and weaker signals than the untreated control (Figure 3A). Analysis by qRT–PCR corroborated immunostaining results: treatment with TGF-β stimulated the expression of *COL1A2* mRNA, and halofuginone was able to attenuate this upregulation, as well as to suppress basal *COL1A2* mRNA expression (Figure 3B).

### Halofuginone exerts its inhibitory effects via down-regulation of Smad3 expression

**Figure 4:**
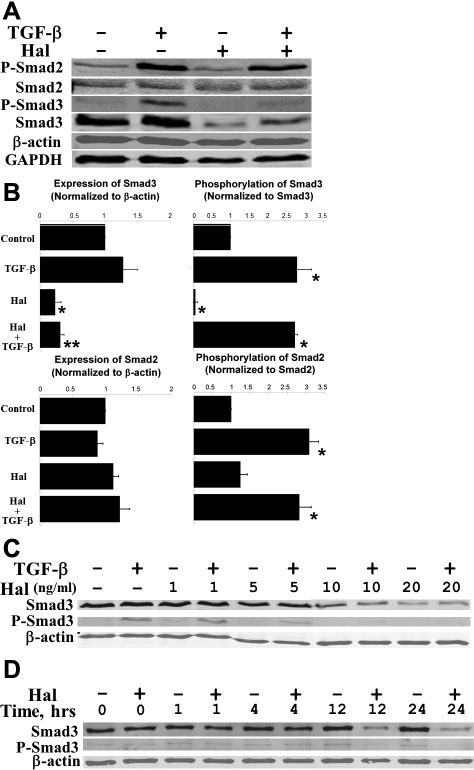
Halofuginone treatment down-regulates Smad3 protein expression but not relative Smad3 phosphorylation in human corneal fibroblasts. **A**: Representative western blots show halofuginone (Hal) reduces Smad3 protein expression. While Smad2 phosphorylation (P-Smad2) is not affected by halofuginone, the intensity of phosphorylated Smad3 (P-Smad3) signal decreases significantly. β-actin and GAPDH shown as simultaneous loading controls. **B**: Quantification and normalization of Smad2, phospho-Smad2, Smad3, and phospho-Smad3 western blot data. * p<0.05 compared to control, ** p<0.05 compared to TGF-β (n=3, bar=S.E.M.). **C**: Dose-dependent down-regulation of Smad3 protein by halofuginone treatment for 24 h before addition of TGF-β. **D**: Time-dependent down-regulation of Smad3 protein by 10 ng/ml halofuginone treatment.

After 24-h treatment with halofuginone, we observed the down-regulation of Smad3 protein in human corneal fibroblasts (Figure 4A). When normalized to β-actin, Smad3 protein in cells treated with halofuginone is significantly less than in either the untreated control or in cells treated only with TGF-β (Figure 4B, upper left panel). In cells treated with halofuginone and TGF-β, the ratio of phosphorylated Smad3 protein to total Smad3 protein is comparable to the ratio in cells treated only with TGF-β (Figure 4B, upper right panel). On the other hand, neither the phosphorylation nor the expression of Smad2 was significantly affected by halofuginone (Figure 4B, lower panels). Figure 4B summarizes results from three independent experiments. The observed effect of halofuginone on total Smad3 expression is both dose- and time-dependent: significant reduction of Smad3 protein is observed only at concentrations of 5 ng/ml or greater (Figure 4C) and only after treatment of 4 h or longer, the greatest effect being seen at 24 h (Figure 4D).

### Halofuginone reduces reporter luciferase activity in transfected corneal fibroblasts

**Figure 5:**
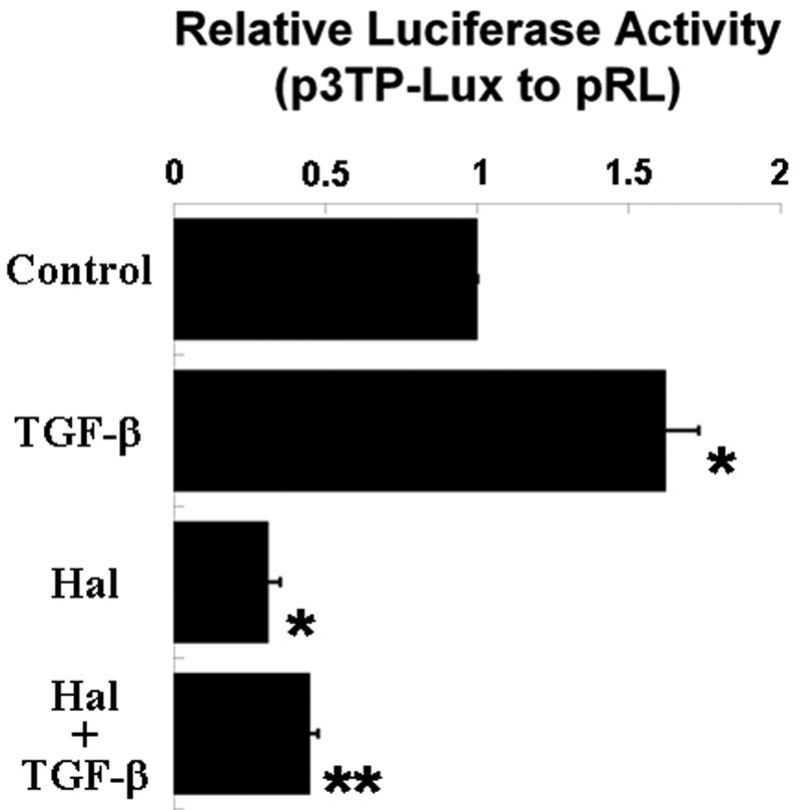
Halofuginone suppresses Smad3-related transcriptional activity in transfected human corneal fibroblasts. Transfected cells treated with TGF-β display significantly greater reporter luciferase activity than untreated control cells, and halofuginone (Hal) significantly inhibits this induced reporter activity. Halofuginone also reduces basal reporter luciferase activity. Reporter luciferase activity expressed as firefly activity (from p3TP-Lux) relative to *Renilla* luciferase activity (from pRL). Results from three independent experiments are shown. * p<0.05 compared to control, ** p<0.05 compared to TGF-β (n=3, bar=S.E.M.).

Because we found halofuginone to decrease Smad3 expression but not relative Smad3 phosphorylation (Figure 4), we investigated endogenous Smad3-related transcriptional activity using a luciferase reporter plasmid, p3TP-Lux. We confirmed that TGF-β treatment significantly increases reporter luciferase activity, as normalized to control luciferase activity, in transfected human corneal fibroblasts (Figure 5). Treatment with halofuginone significantly inhibits this upregulation of reporter luciferase activity, as well as significantly decreases basal reporter luciferase activity in cells not treated with TGF-β (Figure 5).

## Discussion

Smad3 is a key mediator of the TGF-β-depedent fibrotic response [27]. Smad3 signaling has become a promising target for antifibrotic therapies [8], and both genetic and chemical approaches have been developed to disrupt this pathway. Among the chemical agents able to inhibit Smad3 potently, halofuginone is one of the few being studied extensively in humans as well as in animals. However, to our knowledge, the antifibrotic effects of halofuginone on cultured corneal fibroblasts or on the intact cornea have not been studied previously. Here we report halofuginone suppresses the expression of fibrotic markers and ECM proteins in cultured human corneal fibroblasts; using a variety of techniques, we demonstrated that halofuginone treatment significantly reduces the TGF-β-induced expression of fibronectin, α-SMA and type I collagen and formation of stress fiber assemblies (Figure 2 and Figure 3). Based on these findings and its low toxicity on corneal fibroblasts (Figure 1), halofuginone may serve as a promising candidate for reducing corneal fibrosis and haze and restoring vision in patients with corneal injuries or disorders.

These results, i.e., significantly reduced expression of fibrotic markers and ECM proteins by halofuginone, are consistent with halofuginone studies on fibroblasts from other tissues, e.g. from skin. Our results also indicate that, in cultured corneal fibroblasts, the suppression of fibrotic markers and ECM proteins by halofuginone may be mediated by the down-regulation of Smad3 protein expression, which has not been reported previously to our knowledge. This reduced Smad3 protein expression is accompanied by less total phosphorylated Smad3 upon TGF-β stimulation (Figure 4A), which may account for the observed decrease in Smad3-related transcriptional activity as demonstrated by the p3TP-Lux luciferase reporter assay (Figure 5). The observed effect on Smad3 protein expression is dose-dependent (Figure 4C) and most prominent at 24-h treatment (Figure 4D), but can be reversed by withdrawing halofuginone treatment (data not shown).

Our Smad3 results are in contrast to previous reports, which do not report down-regulation of total Smad3 protein but instead indicate that halofuginone disrupts Smad3 phosphorylation. Halofuginone pretreatment ranging from 30 min to 24 h inhibits TGF-β-induced phosphorylation of Smad3 in other cell types [18,22,28,29], abolishing downstream transcriptional activity. For example, phosphorylated Smad3 binds directly to the promoter region of the *COL1A2* gene to activate transcription in embryonic mouse dermal fibroblasts; and halofuginone treatment, by specifically suppressing the phosphorylation of Smad3, reduces the expression of type I collagen [18]. Because TβR-II phophorylates the MH2 domains of both Smad2 and Smad3 indiscriminately, such a specific effect on Smad3 phosphorylation may be mediated via post-Smad phosphorylation regulation as proposed by McGaha et al. and Roffe et al. [18,29]. Although one-hour pretreatment with halofuginone effectively suppresses expression of α-SMA, fibronectin, and type I collagen in our human corneal fibroblasts (Figures 2 and 3), one-hour pretreatment did not inhibit TGF-β-induced phosphorylation of Smad3 in our hands (data not shown). We did observe less phosphorylated Smad3 after 24-h pretreatment with halofuginone; however, since we found this decrease was proportional to the observed decrease in total Smad3 (Figure 4B), we believe halofuginone may act via a novel mechanism in corneal fibroblasts which involves modulation of Smad3 expression.

The underlying mechanism by which halofuginone down-regulates Smad3 protein expression in human corneal fibroblasts awaits further investigation. Halofuginone has been shown to upregulate the expression of Smad7 and down-regulate the expression of TβR-II, which in combination lead to the inactivation of Smad2 and Smad3 signaling in various mouse WT and knockout fibroblasts [22]. While western blots showed that halofuginone did not alter the protein expression of either Smad7 (data not shown) or Smad2 (Figure 4B) in our corneal fibroblasts, potential changes in TβR-II expression may be involved and warrant future experiments. A recent study has shown that halofuginone exerts its anti-proliferative effects in an acute promyelocytic leukemica cell line, NB4, by upregulating the expression of Smad3, as well as other TGF-β signaling molecules [30]; it is worth noting, however, that the effective concentrations were significantly higher (75–200 ng/ml) compared to the dose used in our corneal fibroblast study (10 ng/ml). Other signaling pathways may also regulate the protein expression of Smad3: one study on a mouse epithelial cell line proposed the NFκB and/or nitric oxide synthase pathways as mediators of reduced Smad3 protein expression and altered TGF-β signaling resulting from the overexpression of a regulatory domain of the cystic fibrosis transmembrane conductance regulator [31].

TGF-β signaling is highly dependent on the cell type and cell signaling context. For example, corneal fibroblasts isolated from Smad3 knockout mice fail to transform into myofibroblasts under TGF-β treatment, whereas dermal fibroblasts with Smad3 deficiency retain the capability of myofibroblast transformation [32,33]. However, while Smad3 knockout dermal fibroblasts express less fibronectin than wild-type dermal fibroblasts upon stimulation with TGF-β [2], this induced fibronectin expression is not different between Smad3 heterozygous knockout and Smad3 homozygous knockout corneal fibroblasts [33]. Furthermore, studies on transgenic animals and cultured fibroblasts in vitro show that, although similar in many aspects, the cutaneous and corneal wound healing processes are distinct in regards to the regulation of TGF-β production, the response of inflammatory cells to TGF-β, and the expression of fibrotic markers [2,33]. Corneal fibroblasts may respond to halofuginone differently due to their unique features in TGF-β signaling and the fibrotic process.

In addition to disrupting Smad3 signaling, halofuginone has also been found to activate the PI3K/Akt and ERK pathways in muscle cells [29], activate c-Jun signaling in mouse embryonic fibroblasts [18], inhibit NFκB and p38 MAPK in activated T cells [34], and activate NFκB and p38 MAPK in hepatic stellate cells [35]. As indicated by the above reports and our results, the effects of halofuginone may be highly dependent on the cell type and/or the cellular signaling context. Because halofuginone seems to affect distinct signaling pathways in different cell and tissue types, it may be desirable to avoid systemic administration of halofuginone; for antifibrotic applications, a cell- or tissue-specific delivery method may be needed instead to minimize unwanted side effects.

In summary, data from the present study suggest that halofuginone has the potential to mitigate the deleterious effects of corneal fibrosis. Our findings present a new opportunity for managing fibrosis of the ocular surface by down-regulating Smad3. Future investigations will better establish the signaling pathway through which halofuginone exerts its effects and will examine whether these in vitro results translate to in vivo efficacy.
